# Dose–response evaluation of urinary cadmium and kidney injury biomarkers in Chinese residents and dietary limit standards

**DOI:** 10.1186/s12940-021-00760-9

**Published:** 2021-06-30

**Authors:** Ying Qing, Jiaqi Yang, Yuanshen Zhu, Yongzhen Li, Weiwei Zheng, Min Wu, Gengsheng He

**Affiliations:** 1 School of Public Health/Key Laboratory of Public Health Safety, Ministry of Education, Department of Nutrition and Food Science, Fudan University, No. 130 Dongan Road, Shanghai, 200032 China; 2 Department of Environmental Health, School of Public Health, Fudan University, Shanghai, 200032 China

**Keywords:** Cadmium, Toxicokinetic model, Effect biomarkers, Benchmark dose, Tolerable daily intake

## Abstract

**Background:**

Cadmium (Cd) is a common heavy metal that mainly causes renal damage. There is a lack of research on the large-scale and systematic evaluation of the association between urinary Cd (U-Cd) and various effect biomarkers among Chinese residents.

**Methods:**

Based on the establishment process of dietary Cd limit standards by European Food Safety Authority (EFSA), the dose–response relationships between U-Cd and four biomarkers, β_2_-microglobulin (β_2_-MG), N-acetyl-β-glucosidase (NAG), microalbumin (mALB), and retinol binding Protein (RBP) were explored, respectively. Toxicokinetic model was used to derive the dietary Cd exposure limit for Chinese residents after critical U-Cd concentration was calculated.

**Results:**

As the sensitive biomarkers of renal injury, β_2_-MG and NAG were selected to estimate the 95% confidence interval lower limit of the U-Cd benchmark dose (BMDL_5_) to be 3.07 and 2.98 μg/g Cr, respectively. Dietary Cd exposure limit was calculated to be 0.28 μg/kg bw/day (16.8 μg/day, based on the body weight of 60 kg), which was lower than the average Chinese Cd exposure (30.6 μg/day) by the China National Nutrient and Health Survey.

**Conclusion:**

This study established an overall association between U-Cd and renal injury biomarkers, and explored the Chinese dietary Cd exposure limits, which helps improve Chinese Cd exposure risk assessment and provides a reference basis for formulating reasonable exposure standards.

**Supplementary Information:**

The online version contains supplementary material available at 10.1186/s12940-021-00760-9.

## Introduction

Cadmium (Cd) is a toxic heavy metal commonly found in the environment, with an average concentration in the earth's crust of about 0.2 mg/kg [1]. With the development of social industry, human activities, such as mineral development, metal smelting, industrial emissions, fossil fuels, and waste incineration, have caused Cd to enter soil and water through farmland irrigation, atmospheric dust reduction and urban compost [2, 3] and lead to long-term and wide presence of Cd in the environment. Kidney is the main target organ of Cd exposure [4, 5]. Adverse outcomes such as tubular injury, decreased glomerular reabsorption rate have been shown to be closely related to Cd exposure [6].

Considering the potential hazards of Cd exposure to human health, the European Food Safety Authority (EFSA) proposed a tolerable weakly intake (TWI) of 2.5 μg/kg bw/week for dietary Cd intake in 2009 [7]. The committee used a meta-analysis to determine the dose–response relationship between urinary Cd (U-Cd) and the renal injury biomarker β_2_-microglobulin (β_2_-MG). Benchmark dose lower-bound confidence limit (BMDL) of U-Cd, which was derived by Hill model, was used as the safety threshold dose. Based on the critical U-Cd concentration, corresponding external exposure limit was calculated by toxicokinetic (TK) model. The TK model is an in vitro mathematical model based on physiology, biochemistry, dissection and pharmacokinetics, which could simulate the internal exposure dose in specific organs based on the external exposure of pollutants, and provide reliable information for quantitative assessment of the metabolic concentrations of toxic chemicals [8]. At present, it has been widely used in the risk assessment of pollutants such as heavy metals, persistent organic pollutants, and organic pesticides [9–11].

By integrating and reanalyzing the published data, a comprehensive meta-analysis can reduce the uncertainty in a single study and improve the representativeness of the analysis results [12]. This is also a commonly used method for establishing pollutant limit standards and safety thresholds and risk assessments internationally [7, 10]. However, China has not established dietary Cd exposure limits based on the exposure characteristics of Chinese. In previous risk assessment studies, international standards such as EFSA or the Joint FAO/WHO Expert Committee on Food Additives (JECFA) standards were often used to evaluate the risk of the Chinese residents. The Cd pollution status and dietary patterns of the main data sources of the above standards, such as Europe and Japan, are quite different from those of China, inevitably affecting the accuracy of Cd exposure risk assessment in China. Therefore, it is essential to investigate the sensitive effect biomarker of Cd exposure and reference dietary Cd intake in Chinese population.

This study is based on the epidemiological studies of U-Cd and renal injury in China, aiming to evaluate the effects of Cd exposure on renal biomarkers in Chinese. In particular, this study attempts to (1) establish a dose–response relationship between U-Cd and renal biomarkers; (2) calculate the critical threshold dose of U-Cd in Chinese population based on continuous data; (3) use the TK model to calculate the dietary Cd exposure limit applicable to the Chinese population. As far as we know, this is the first study to conduct a large-scale systematic analysis of U-Cd and multiple effect biomarkers and estimate the tolerable dietary intake (TDI) limit of Cd in China. The results will provide reliable information for the establishment of dietary Cd limits and improve the risk assessment of Cd exposure among Chinese.

## Materials and methods

### Data collection

In this study, renal injury effects were used as the endpoints of Cd exposure. Systematic screening was performed in four common databases (Web of Science, PubMed, China National Knowledge Infrastructure (CNKI) and WANFANG Database). The U-Cd with renal injury biomarkers in Chinese were used as the research object to establish a comprehensive database.

Based on previous researches, four renal injury biomarkers were selected: β_2_-MG, N-acetyl-β-glucosidase (NAG), microalbumin (mALB), and retinol binding Protein (RBP). Taking the β_2_-MG as an example, the following search formulas were used to search in the databases: PubMed: Cadmium AND β_2_-Microglobulin AND (China OR Hongkong OR Taiwan); Web of science: TS = (Cadmium AND β_2_-Microglobulin) AND TS = (China OR Hongkong OR Taiwan); CNKI Database: SU = Cadmium AND SU = β_2_-Microglobulin; WANFANG Database: Subject: (Cadmium)*Subject: (β_2_-Microglobulin). (Published time: 1980.1–2021.3).

Literatures were included in a consolidated database by detailed screening based on the following criteria: 1. The study measured U-Cd (in μg/g creatinine or other units that could be converted) as biomarker of internal dose together with at least one biomarker of renal injury both as continuous variables; 2. If the research data are used in more than one study, the study providing the most complete and detailed information was chosen (e.g., the study which provides the most dose sub-groups); 3. In order to reduce the heterogeneities, the outliers and sample size below 15 were excluded.

### Data processing

In order to harmonize and validate the database, some further checks and transformations were performed. Abnormal values or data from the same population were double-checked. The units of U-Cd were adjusted in consistent. U-Cd values reported in nmol/mmol Cr were transformed into μg/g Cr by applying the factor of 0.99375 (= Molar Mass (Cd)/ Molar Mass (creatinine)) [13]. In addition, geometric mean (GM) and geometric standard deviation (GSD) were used to record the included data. If the study only provided the arithmetic mean (AM) and arithmetic standard deviation (SD), the following data transformations were made [14, 15]:

1 $$GM=AM/\sqrt{(1+({SD/AM)}^{2}})$$

2 $$GSD=exp[\sqrt{log(1+{\left(SD/AM\right)}^{2})}]$$

If only the median and range (min, max) were provided, GM and GSD were converted according to the following formula [16]:

3 $$GM = Median$$

4 $$GSD={e }^{max(\mathit{log}\left(GM/min\right), log(max/GM)/{}^{-1}(1-1/2n)}$$

where *Φ* represents the cumulative density function of the standardized Gaussian distribution and *n* represents the sample size.

### Calculation of U-Cd safety threshold

After the databases compilation, the benchmark dose software (BMDS) was used to calculate the BMD and BMDL. The Hill model (based on the EFSA, as preferred) and exponential model (as complementary) were used to evaluate the dose–response relationship between the mean dose of each population subgroup to the mean response. Akaike's information criterion was performed for model selection, and the model with the lowest Akaike's information criterion value was preferred. Goodness of fit test was conducted with the criteria of *P* > 0.05. The benchmark response factor (BMRF) is set at 5%. The risk type of BMR is extra for Hill model, indicating that the response associated with the BMR is the background estimate plus the product of the BMRF times the difference between the background estimate and the model estimates of the maximum response. The BMR type for exponential model is standard deviation (Std. Dev.), indicating the response associated with the BMR is the background estimate plus the product of the BMRF times the standard deviation for the control group data [17]. Among 4 biomarker databases, the BMD and BMDL of U-Cd were calculated and compared, and the lowest dose was selected as the safety threshold.

The dose–effect relationship described by Hill model is an S-shape with 4 parameters. The model equation is given by:

5 $$Effect\left(d\right)=background+amplitude\times {(d}^{\mathrm{\eta }}/({d}^{\mathrm{\eta }}+{ed}_{50}^{\mathrm{\eta }}))$$

where *d* stands for the dose (U-Cd), ‘amplitude’ corresponds to the difference between the 2 plateaux of the S-shape, ed50 corresponds to the dose where 50% of the maximal effect is achieved, and eta (η) corresponds to the shape parameter defining the steepness of the S curve. The exponential model, as a complementary model, is also used to fit the dose–effect relationship when the hill model does not fit well.

### Derivation of dietary Cd expose TDI

Based on the U-Cd safety threshold, the TK model was used to estimate the TDI limits of the dietary Cd exposure. The formula of the TK model is as follows [18]:

6 $${Cd}_{urine}\left(age\right)=\frac{{f}_{u}\times {f}_{k}}{\mathrm{l}\mathrm{o}\mathrm{g}(2)}\times d\times {t}_{1/2}\frac{[1-\mathrm{e}\mathrm{x}\mathrm{p}(-\frac{\mathrm{log}\left(2\right)\times age}{{t}_{1/2}})]}{[1-\mathrm{e}\mathrm{x}\mathrm{p}(-\frac{\mathrm{log}\left(2\right)}{{t}_{1/2}})]}$$

where *Cd*_*urine*_* (age)* represents the U-Cd (μg/g Cr) at specific age; *d* is daily dietary Cd exposure (μg/kg/day); *t*_*1/2*_ is the half-life of Cd in the renal; *f*_u_*f_*k*_ represents the comprehensive constant related to absorption. Parameters of the model referred to EFSA which were as follows [7, 18]: the half-life was drawn from a lognormal distribution (Mean: 11.6, SD: 3.0) to consider the variability among populations; *age* was set to be 50 years old; the absorption rate (*f*_u_*f_*k*_) is fixed at 0.0063. In addition, considering the differences between the studies, adjustment factor (AF^(95)^ = 3.9) was introduced to account for the lack of individual data and avoid overestimation of the TDI limit [7]. A total of 100,000 Monte Carlo sampling iterations was conducted to estimate the relative frequency distribution of dietary Cd exposure in the Chinese corresponding to the U-Cd at threshold dose using @Risk. To ensure that 95% of the residents' U-Cd are below the safety threshold, the dietary Cd exposure corresponding to 5^th^ percentiles of the population reaching this threshold was selected as the TDI limit.

### Statistical analysis

The BMD/BMDL was calculated by BMDS (Version 2.7.0, Environmental Protection Agency, USA; https://www.epa.gov/bmds/benchmark-dose-software-bmds-version-27-materials). @Risk 7.6 (Palisade software, USA) was used to conduct Monte Carlo sampling. Origin 2018 (OriginLab, Northampton, Massachusetts, USA) was used for drawing. SPSS 16.0 (SPSS Inc., Chicago, IL, USA) was used to perform other statistical analysis. Spearman correlation was used to determine the association between U-Cd and renal effect biomarkers. Mann–Whitney U test was used to compare the U-Cd or biomarkers among different subgroups. *P* < 0.05 was considered as statistically significant.

## Results

### Included studies

**Fig. 1:**
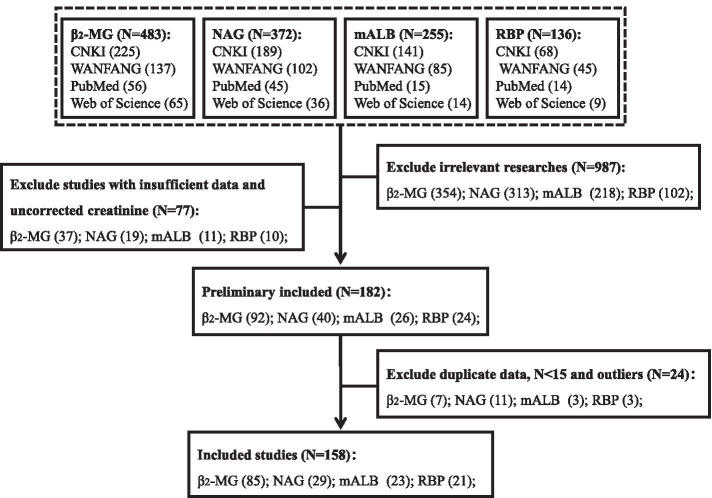
Retrieval and exclusion flow chart of U-Cd and renal injury biomarkers

After searching from 4 databases, a total of 158 studies were included in this study. The process of inclusion and exclusion of the study is shown in Fig. 1. Of all the included studies, 332 matched pairs of U-Cd and β_2_-MG levels can be gathered from 85 studies, followed by NAG (N = 29 with 143 data), mALB (N = 23 with 87 data), and RBP (N = 21 with 74 data). All included publications are listed in the supplementary material (Table S3-6). The composition of gender and occupational status in the database of different effect biomarkers is detailed in the supplementary material (Table S1). The gender composition of each dataset is uniform, and the sample size of the non-occupational is higher than that of the occupational population.

### Characteristics of U-Cd and four effect biomarkers

**Fig. 2:**
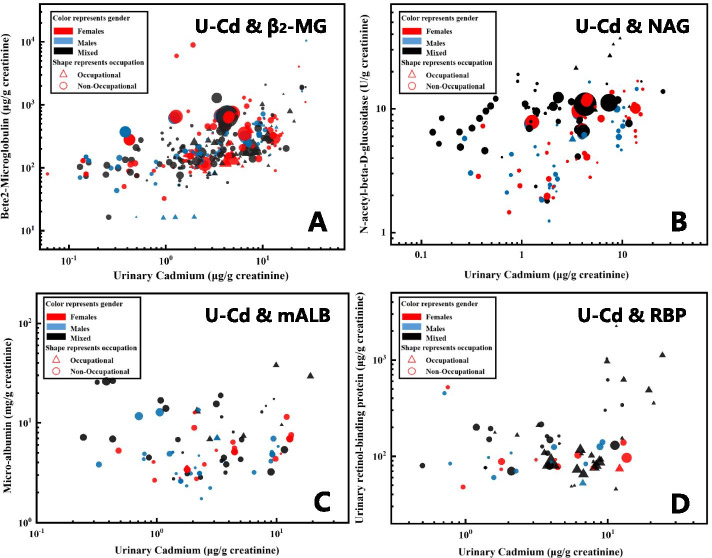
The scatter plot for the U-Cd and renal effect biomarkers (Color represents gender: red (female); blue (male), black (gender information is not clear); shape represents occupational: round (non-occupational), triangle (occupational); dot size represents sample size: size = sqrt (sample size)/158)

The characteristics of U-Cd and four corresponding biomarkers (β_2_-MG, NAG, mALB, and RBP) in each dataset are listed in Table 1. In the database of β_2_-MG, the mean values of U-Cd and β_2_-MG are 4.89 and 384.6 μg/g Cr. In the NAG database, the mean values of U-Cd and NAG are 4.43 μg/g Cr and 8.18 U/g Cr. In the mALB databases, the mean values of U-Cd and mALB are 4.23 μg/g Cr and 7.66 mg/g Cr. In the database of RBP, the mean values of U-Cd and RBP are 6.12 and 215.62 μg/g Cr, respectively. Spearman coefficients show that β_2_-MG and NAG have significant positive correlations with U-Cd. The distribution characteristics of age, gender, and occupational exposure subgroups are listed in the supplementary material (Table S2). Figure 2 depicts the scatter plot of the U-Cd and four effect biomarkers on the log–log scale. Four biomarkers all show an increasing trend with U-Cd and the trend is more pronounced in high concentrations range.

**Table 1:** Dataset characteristics and Spearman coefficients of U-Cd and different effect biomarkers

Dose–response	Mean (SD)	Median	*P*_10_-*P*_90_	Correlation Coefficient
**U-Cd & β**_**2**_**-MG (*N* = 332)**				
U-Cd (μg/g Cr)	4.89 (5.06)	3.29	0.48–11.54	0.569^*^
β_2_-MG (μg/g Cr)	384.57 (872.81)	197.90	81.85–653.23	
**U-Cd & NAG (*N* = 143)**				
U-Cd (μg/g Cr)	4.43 (4.45)	2.38	0.43–11.59	0.398^*^
NAG (U/g Cr)	8.18 (5.56)	7.64	2.21–14.02	
**U-Cd & mALB (*N* = 87)**				
U-Cd (μg/g Cr)	4.23 (3.95)	2.49	0.77–10.41	0.164
mALB (mg/g Cr)	7.66 (6.67)	5.10	2.79–15.78	
**U-Cd & RBP (*N* = 74)**				
U-Cd (μg/g Cr)	6.12 (4.96)	4.56	0.87–12.60	0.146
RBP (μg/g Cr)	215.62 (322.58)	107.92	67.51–503.00	

^*^: *P* < 0.05

### Urinary Cd safety threshold

**Fig. 3:**
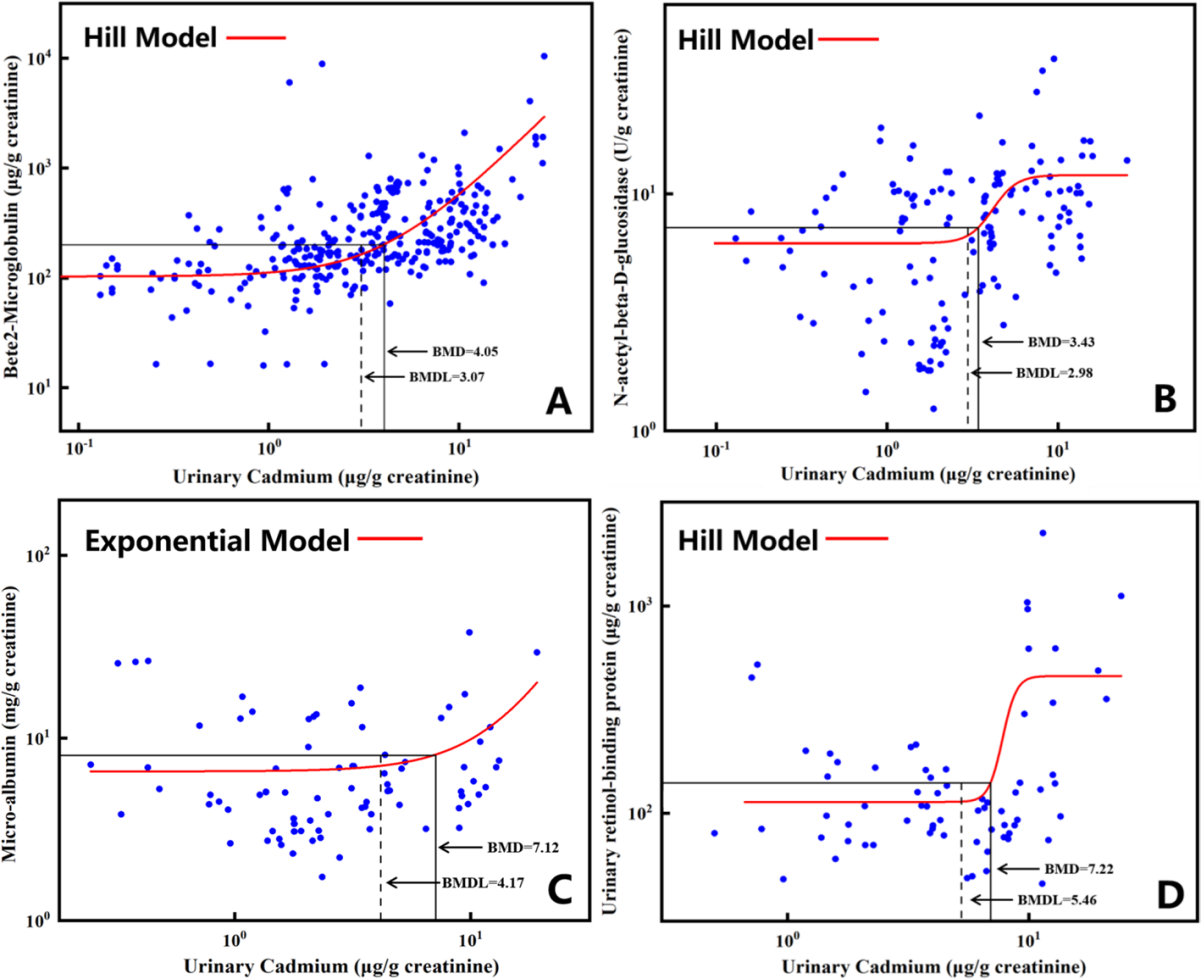
Dose–response relationship between U-Cd and effect biomarkers. (The red solid curve is a fitted dose–response model (Hill or Exponential model). The horizontal line corresponds to a 5% change of the range of the response in the background (BMR = 0.05). The intersections of the horizontal line and the curve is the BMD. The BMDL is illustrated by the dashed-dotted line and represents the lower limit of the 95% confidence interval for BMD.)

The dose–response relationship fit by Hill or exponential model of U-Cd and the four effect biomarkers are shown in Fig. 3. Except for mALB, which uses the exponential model for fitting, the other three effect biomarkers are all fitted by Hill model. For the four effect biomarkers of β_2_-MG, NAG, mALB and RBP, the calculated BMD_5_ is 4.05, 3.43, 7.12 and 7.22 μg/g Cr, respectively, and the BMDL_5_ is 3.07, 2.98, 4.17 and 5.46 μg/g Cr, respectively. The corresponding U-Cd safety thresholds are listed in Table 2. The threshold dose of U-Cd derived by β_2_-MG and NAG are lower than the results of the mALB and RBP. EFSA chose β_2_-MG as the key biomarker, while NAG is the most sensitive biomarker in this study. Therefore, β_2_-MG and NAG were both selected as sensitive biomarkers for comparison. The corresponding BMDL_5_ (β_2_-MG: 3.07 μg/g Cr; NAG: 2.98 μg/g Cr) were used as U-Cd safety thresholds to derive the tolerable dietary Cd intake limits, respectively.

**Table 2:** The BMD_5_/BMDL_5_ of the U-Cd for renal injury biomarkers

Biomarkers	BMD model	BMD_5_ (μg/g Cr)	BMDL_5_ (μg/g Cr)
β_2_-MG	Hill	4.05	3.07
NAG	Hill	3.43	2.98
mALB	Exponential	7.12	4.17
RBP	Hill	7.22	5.46

### Tolerable dietary intake for Cd exposure

**Fig. 4:**
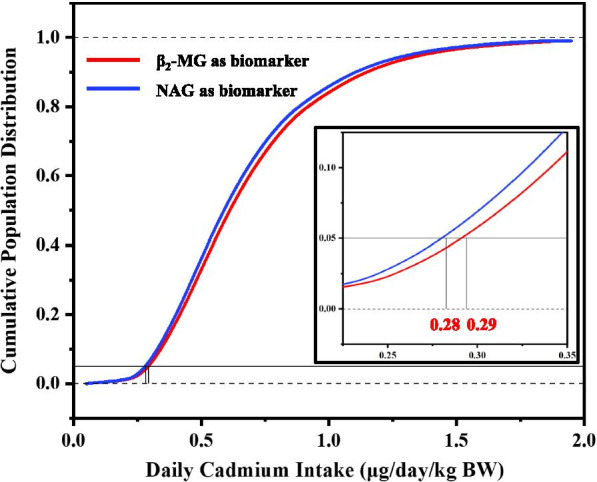
Cumulative population frequency distribution of daily dietary Cd expose considering NAG and β2-MG as biomarkers to calculate U-Cd threshold

The cumulative probability distribution of dietary Cd exposure in Chinese population derived from the BMDL_5_ of U-Cd of NAG and β_2_-MG is shown in Fig. 4. In order to protect most people from kidney injury caused by Cd exposure, the dietary intake corresponding to the 5% quantile is selected as the TDI value, which are 0.28 μg/kg bw/day for NAG and 0.29 μg/kg bw/day for β_2_-MG, respectively. The more stringent value (0.28 μg/kg bw/day) was decided as the final TDI limit (16.8 μg/day, based on body weight of 60 kg). Within this TDI, at least 95% of the population have U-Cd concentrations below the safe threshold.

## Discussion

This study focused on the U-Cd and renal injury biomarkers among Chinese and conducted a comprehensive search for related studies. The dose–response relationships between U-Cd and four renal injury biomarkers were established using Hill or exponential model. β_2_-MG and NAG were used as the sensitive biomarkers to determine the BMDL_5_ of U-Cd safe thresholds (β_2_-MG: 3.07 μg/g Cr; NAG: 2.98 μg/g Cr). The TDI limit derived by TK model was determined to be 0.28 μg/kg bw/day. Within this limit, U-Cd concentrations of 95% of the residents in China would be below the safety threshold, effectively avoiding the risk of renal damage caused by dietary Cd exposure. This study provides reference for formulating of China's Cd exposure standards, which is conducive to objectively and accurately assessing the Cd exposure health risk of Chinese residents.

The dose–response relationship is the basis for risk assessment of pollutant exposure. In the past, the “No-observed-adverse-effect-level” (NOAEL), usually obtained by animal experiments, was commonly served as the potential key dose endpoint related to human health [19]. However, the dose setting, the sensitivity of the detection method and the sample size in the experimental design will have a great impact on the NOAEL results in practice. The BMD method, which was first proposed by Crump in 1984 [20], has been widely adopted in health risk assessment. The BMD is defined as the exposure level corresponding to a predetermined increase in the probability of an adverse response (e.g., 1%–10%) above the background level [21]. Because BMD can make full use of dose–response data and has lower dependence on experimental dose, which increases the reliability and accuracy of the results, it has been widely used in risk assessment [22].

U-Cd is the main indicator reflecting the degree of Cd accumulation in the body under long-term exposure, while blood Cd represents the Cd that enters the body more recently [23]. Choosing U-Cd as the internal exposure marker can well reflect the effect of long-term Cd exposure on the renal injury biomarkers. Renal injury is the main toxic effect caused by chronic low-dose Cd exposure [24]. β_2_-MG, RBP and NAG are biomarkers of renal tubular injury, and mALB is a biomarker of renal glomerular insufficiency [25]. This study showed that β_2_-MG and NAG were more sensitive to the changes in U-Cd. This suggested that tubular biomarkers were more sensitive to environmental Cd exposure than glomerular biomarkers, which was consistent with previous research [25]. However, the correlation between U-Cd and biomarkers of kidney function will be affected by many aspects such as normal variation in renal function (including changes in urinary flow) and concentration adjustment methods (creatinine, specific gravity, etc.). Low-level urinary Cd corrected with creatinine may cause a false association between Cd exposure and protein excretion [26]. Moreover, it is still inconclusive whether β_2_-MG or NAG can better reflect the degree of kidney injury. An 8-year follow-up study conducted in China's Cd-contaminated area showed that monitoring β_2_-MG can provide more comprehensive information on renal tubular function changes than NAG [27]. However, Moriguchi J et al. found that the correlation coefficient between U-Cd and NAG was higher than β_2_-MG, and NAG could be used as the most sensitive biomarker for monitoring renal tubular dysfunction for residents in non-polluted areas [28].

The safety threshold of U-Cd in Chinese (2.98 μg/g Cr) obtained in this study is lower than the EFSA result (4 μg/g Cr) [14], which was based on the U-Cd and β_2_-MG data in populations around the world. This could be caused by the fact that the data of EFSA contained extremely high values (β_2_-MG > 100,000 μg/g Cr) and affected the overall distribution of the model, resulting in higher BMDL. Other studies also supported a relative lower threshold of U-Cd. A recent evaluation by the International Union on Pure and Applied Chemistry (IUPAC) estimated a LOAEL for renal dysfunction of 2 nmol/mmol Cr (2 μg/g Cr) [29]. A study in China explored U-Cd limits in a polluted area in southwest China and determined BMDL_5_ to be 3.48 μg/g Cr [30]. The BMDL_5_ of U-Cd based on β_2_-MG in non-polluted areas in Zhejiang, China, was calculated to be 0.62–0.64 μg/g Cr [31]. Another study of residents in five different regions of China calculated the BMDL_10_ of U-Cd based on β_2_-MG to be 1.69–2.00 μg/g Cr [32]. Different characteristics of Cd exposure, pollution levels, and BMD analysis methods could lead to different BMDL results. The data of this study comes from a systematic analysis of nationwide researches, which includes not only ordinary residents, but also some polluted areas and occupationally exposed people. The obtained U-Cd safety threshold could cover most areas of China and represent the average level of Chinese population.

The TDI value of Cd in this study (16.8 μg/day for a 60 kg BW of adults) was lower than that of EFSA (21.6 μg/day) [7] and JECFA (50 μg/day) [33]. However, some cohort studies suggested that even if the dietary Cd intake was below the EFSA or JECFA standards, it might still increase the risk of death from cancer, cardiovascular disease, and Alzheimer's disease [34, 35]. A Swedish cohort study showed that a 32% increased risk of osteoporosis and 31% increased risk of fracture were observed when dietary Cd exposure was above 13 μg/day [36]. The impairment caused by long-term and low-level dietary Cd intake in population has now been implicated in more serious health outcomes than previously perceived [37]. This study referred to the EFSA standard establishment process and used BMDL_5_ and 5% population risk, which was a very conservative standard.

According to China National Nutrient and Health Survey, the mean Cd exposure of the general population was 15.3 μg/kg bw/month (approximately 30.6 μg/day), which was higher than the TDI in this study. Rice (contribution rate of over 55.8%), leafy vegetables (11.8%) and wheat flour (10.5%) were the three most important contributors to dietary Cd exposure. For the high exposure sub-population with Cd exposure above the 95^th^ percentile, rice was still the main contributor (58.6%), followed by shellfish (13.2%) and leafy vegetables (9.2%) [38]. Another study in non-polluted areas of China pointed out that the median lifetime cumulative Cd intakes was 0.5 g [39], higher than the result from this study (0.37 g, calculated based on age 60). Due to the persistent of Cd in the environment and its high transfer rate from soil to plant [40], maintenance of low Cd in crops and leafy is pivotal. The more stringent TDI value of Cd put forth in this study suggested the need for a revision of dietary standards of Cd and public measures to minimize the food-chain contamination in China.

This study is the first to explore dietary limits for Chinese population based on a systematic analysis of four effect biomarkers of Cd exposure, but it is still subject to following limitations. First, the included research data were from different laboratories. The detection methods of U-Cd and effect biomarkers were inconsistent, which might cause systematic errors. Second, due to the low number of studies on other effect biomarkers (α_1_-microglobulin, bone density, serum calcium, etc.), only four renal injury biomarkers were analyzed. More high-quality and large-scale epidemiological studies in the future are warranted to provide reliable data for sensitive effect biomarkers and health risk assessment. Third, the TK model used to derive the TDI is established based on a Swedish female cohort. Due to the regional and ethnic differences in the absorption and metabolism characteristics of Cd in Chinese and European, the uncertainty of the results obtained by this model increased [41]. Last, potential kidney function damage and adjustment approach of biomarker concentrations in urine samples may affect the association between U-Cd and biomarkers of kidney function [42]. As there are few related prospective studies, it has become an inevitable obstacle to assess the causal relationship between pollutants and adverse outcomes in environmental epidemiological research.

## Conclusions

This study comprehensively analyzed the dose–response relationship between U-Cd and renal injury biomarkers in Chinese residents. The BMDL_5_ of U-Cd using β_2_-MG and NAG as sensitive biomarkers was taken as the critical threshold of Cd exposure. Based on the TK model, a preliminary discussion on the limit standards of dietary Cd exposure for residents in China was conducted. This study provides a reference for formulating Chinese dietary Cd exposure limit standards.

## Supplementary Information

**Additional file 1: Table S1**. Composition of gender and occupation sample size (absolute values and percentage) for different kidney injury indicators. **Table S2**. Data distribution characteristics of age, gender, and occupational exposure subgroups of U-Cd and responder in different data sets. **Table S3**. Study-ID and corresponding references for urinary cadmium (U-Cd) and β2-microglobulin (β2-MG). **Table S4**. Study-ID and corresponding references for urinary cadmium (U-Cd) and N-acetyl-β-glucosidase (NAG).**Table S5**. Study-ID and corresponding references for urinary cadmium (U-Cd) and microalbumin (mALB). **Table S6**. Study-ID and corresponding references for urinary cadmium (U-Cd) and retinol binding Protein (RBP).

## Data Availability

The datasets used and/or analyzed during the current study are available from the corresponding author on reasonable request.

## Abbreviations

Cd: Cadmium
Cr: Creatinine
U-Cd: Urinary Cd
β_2_-MG: β_2_-Microglobulin
NAG: N-acetyl-β-glucosidase
mALB: Microalbumin
RBP: Retinol binding Protein
TWI: Tolerable weakly intake
TDI: Tolerable dietary intake
BMD: Benchmark dose
BMDL: Lower confidence bounds of benchmark dose
BMR: Benchmark response
TK: Toxicokinetic
NOAEL: No-observed-adverse-effect-level
LOAEL: Low-observed-adverse-effect level
