# Supplementary material for: Dose–response evaluation of urinary cadmium and kidney injury biomarkers in Chinese residents and dietary limit standards
Source: Environ Health. 2021 Jun 30;20:75. doi: 10.1186/s12940-021-00760-9 (PMC8247151; doi:10.1186/s12940-021-00760-9)
Supplement: Supplementary file 1 — Additional file 1: Table S1. Composition of gender and occupation sample size (absolute values and percentage) for different kidney injury indicators. Table S2. Data distribution characteristics of age, gender, and occupational exposure subgroups of U-Cd and responder in different data sets. Table S3. Study-ID and corresponding references for urinary cadmium (U-Cd) and β2-microglobulin (β2-MG). Table S4. Study-ID and corresponding references for urinary cadmium (U-Cd) and N-acetyl-β-glucosidase (NAG).Table S5. Study-ID and corresponding references for urinary cadmium (U-Cd) and microalbumin (mALB). Table S6. Study-ID and corresponding references for urinary cadmium (U-Cd) and retinol binding Protein (RBP). [file 12940_2021_760_MOESM1_ESM.docx]

**Supplementary Material**

**for**

**Dose-response evaluation of urinary cadmium and kidney injury biomarkers in Chinese residents and dietary limit standards**

***Table S1.*** Composition of gender and occupation sample size (absolute values and percentage) for different kidney injury indicators

| **Biomarkers** | **N** | **Gender** |  | **Worker’s status** |
| --- | --- | --- | --- | --- |
|  |  | **Males：Females：Mixed** |  | **Worker：Non-Worker** |
| β_2_-MG | 42578 | 10362: 15879: 16337  (24%: 37%: 39%) |  | 7362：35216  (17%: 83%) |
| NAG | 18041 | 4145：5435：8461  (23%: 30%: 47%) |  | 325：17716  (2%: 98%) |
| mALB | 8293 | 2468：2258：3567  (30%: 27%: 43%) |  | 716：7577  (9%: 91%) |
| RBP | 8026 | 1046：1540：5440  (13%: 19%: 68%) |  | 3494：4532  (44%: 56%) |

***Table S2.*** Data distribution characteristics of age, gender, and occupational exposure subgroups of U-Cd and responder in different data sets

| **Dose-response** **^a^** | **Age** | | |  | **Gender** | | |  | **Occupational exposure** | | |
| --- | --- | --- | --- | --- | --- | --- | --- | --- | --- | --- | --- |
|  | **≤ 50** | **> 50** | ***P-value^b^*** |  | **Male** | **Female** | ***P-value^b^*** |  | **Yes** | **No** | ***P-value^b^*** |
| **U-Cd & β2-MG** |  |  |  |  |  |  |  |  |  |  |  |
| *N* **^c^** | 144 | 85 |  |  | 85 | 100 |  |  | 74 | 234 |  |
| U-Cd (μg/g Cr) | 4.7±4.3 | 4.1±3.2 | 0.95 |  | 4.4±4.9 | 5.2±5.1 | 0.15 |  | 6.5±4.2 | 4.4±5.2 | **<0.05** |
| β2-MG (μg/g Cr) | 320.7±891.0 | 375.5±220.3 | **<0.05** |  | 397.1±1133.1 | 508.2±1117.7 | 0.19 |  | 243.7±216.4 | 426.4±983.6 | 0.24 |
| **U-Cd & NAG** |  |  |  |  |  |  |  |  |  |  |  |
| *N* | 40 | 56 |  |  | 42 | 46 |  |  | 7 | 136 |  |
| U-Cd (μg/g Cr) | 4.3±4.4 | 3.8±3.2 | 0.49 |  | 4.2±3.6 | 5.6±4.9 | 0.23 |  | 7.0±3.2 | 4.3±4.5 | **<0.05** |
| NAG (U/g Cr) | 7.2±5.1 | 9.6±5.6 | **<0.05** |  | 6.2±3.9 | 6.7±4.1 | 0.55 |  | 20.7±12.3 | 7.5±4.2 | **<0.05** |
| **U-Cd & mALB** |  |  |  |  |  |  |  |  |  |  |  |
| *N* | 18 | 27 |  |  | 30 | 19 |  |  | 8 | 79 |  |
| U-Cd (μg/g Cr) | 6.1±5.1 | 3.7±3.2 | 0.17 |  | 3.5±3.2 | 5.0±4.5 | 0.17 |  | 7.1±5.9 | 3.9±3.6 | **<0.05** |
| mALB (mg/g Cr) | 11.4±9.2 | 7.1±7.7 | **<0.05** |  | 5.0±2.9 | 5.6±2.7 | 0.19 |  | 14.8±12.1 | 6.9±5.5 | **<0.05** |
| **U-Cd & RBP** |  |  |  |  |  |  |  |  |  |  |  |
| *N* | 39 | 15 |  |  | 13 | 13 |  |  | 30 | 44 |  |
| U-Cd (μg/g Cr) | 6.9±5.6 | 6.4±4.1 | 0.87 |  | 3.8±3.2 | 5.4±4.8 | 0.46 |  | 8.9±5.2 | 4.2±3.8 | **<0.05** |
| RBP (μg/g Cr) | 212.8±240.6 | 177.4±144.7 | 0.72 |  | 131.7±105.9 | 136.1±129.9 | 0.74 |  | 302.3±476.0 | 156.5±122.5 | 0.79 |

**^a^**: Mean ± SD; **^b^**: *P-value* for Mann-Whitney Test; **^c^**: The number of data sets;

***Table S3*.** Study-ID and corresponding references for urinary cadmium (U-Cd) and β_2_-microglobulin (β_2_-MG).

| Study-ID | First author | Journal and Volume number |
| --- | --- | --- |
| 1 | Qiu Yi | Journal of Environment and Health. 2012, 29(6); 549-550 |
| 2 | Xie Ruilin | Journal of occupational health and damage. 2005, 20(2); 90-91 |
| 3 | Li Jimeng | Practical Preventive Medicine. 2018, 25(12); 1438-1440 |
| 4 | Liang Jinxia | Chinese journal of modern drug application. 2010, 04(13); 245-246 |
| 5 | Chen Caihui | China tropical medicine. 2006, 6(6); 958-959 |
| 6 | Yue Weimei | Qingdao medical journal. 2008, 40(3); 222-223 |
| 7 | Li Jimeng | Chinese journal of industrial hygiene and occupational diseases. 2011,29(8); 600-602 |
| 8 | Wang Wenlin | Chinese journal of industrial medicine. 2001, 14(4); 193-196 |
| 9 | Zhou Ni | Modern Preventive Medicine. 2013, 40(8); 1485-1486 |
| 10 | Liang Shunhua | China occupational medicine. 2009, 36(6); 476-477 |
| 11 | Shi Xinshan | China occupational medicine. 2010, 37(1); 49-50 |
| 12 | Huang Bo | Journal of labour medicine. 2000, 17(2);78-80 |
| 13 | Zhang Wenli | Journal of Hygiene Research. 2015, 44(5); 780-787 |
| 14 | Sun Hong | Jiangsu Journal of Preventive Medicine. 2017, 28(4); 361-365 |
| 15 | Wu Ye | Occupation and Health. 2016, 32(7); 960-962 |
| 16 | Li Yanfei | Guangxi Medical Journal. 2016, 38(4); 514-516 |
| 17 | Xie Ruilin | China tropical medicine. 2008, 8(4); 550-551 |
| 18 | Wen Xianzhong | The Journal of practical medicine. 2011, 27(15); 2751-2753 |
| 19 | Wang Renqun | Journal of environment and health. 2006, 23(3); 202-204 |
| 20 | Wang Ting | Guangdong Preventive Medicine Annual Conference. 2011 |
| 21 | Jin Taiyi | Chinese journal of environmental & occupational medicine. 2002, 19(1); 10-16 |
| 22 | Yang Rongxin | Studies of trace elements and health. 2006, 23(6); 41-44 |
| 23 | Chen Guimei | Practical Preventive Medicine. 2016, 23(9); 1147-1149 |
| 24 | Wang Haiyan | Chinese journal of public health. 2008, 24(6); 641-643 |
| 25 | Huang Wenguang | Journal of Environment and Health. 1992, 9(4); 166-168 |
| 26 | Jiang Xiaohong | Occupational health and emergency rescue. 2010, 28(1); 37-40 |
| 27 | Wang Keyue | Journal of Environmental & Occupational Medicine. 2011, 28(12); 738-740 |
| 28 | Chen Lianguang | Occupation and Health. 2013, 29(16); 1972-1973 |
| 29 | Chen Liang | Chinese journal of industrial hygiene and occupational diseases. 2006,24(1); 7-11 |
| 30 | Hong Feng | Chinese journal of preventive medicine. 2004, 38(6); 374-378 |
| 31 | Kong Qinghu | Trace elements science. 1997, 31-36 |
| 32 | Cai Shiwen | Health research. 1989, 18(5); 22-25 |
| 33 | Chen Min | Chinese journal of health laboratory technology. 2008, 18(8); 1535-1536 |
| 34 | Ma Wei | South China Journal of Preventive Medicine. 2017, 43(4); 384-386 |
| 35 | Li Jiqiang | China public health. 2001, 17(6); 486-488 |
| 36 | Wang Zaijuan | Journal of Zhejiang Academy of Medical Sciences. 2001, 1(45); 104-108 |
| 37 | A Lata | Chinese Journal of Industrial Medicine. 1992, 5(1); 8-9 |
| 38 | Shang Qi | Journal of environment and health. 1995; 1-3 |
| 39 | Li Jiangjun | Chinese Journal of Industrial Medicine. 2016, 29(1); 67-68 |
| 40 | Wang Keyue | Journal of occupational health and damage. 2000; 9-10 |
| 41 | A Lata | Industrial Hygiene and Occupational Diseases. 1988, 14(1); 23-24 |
| 42 | Chen Jixiang | China public health. 1994; 502-503 |
| 43 | Zhou Xiaojuan | Chinese Journal of Modern Medicine. 1994, 4(2); 55-56 |
| 44 | Zhang Jieying | Journal of Environmental Hygiene. 2015, 5(1); 39-43 |
| 45 | Li Hao | Journal of Public Health and Preventive Medicine. 2014, 25(6); 13-16 |
| 46 | Liu Zhanqi | China public health. 2001, 17(10); 923-924 |
| 47 | Huang Jie | Chinese Journal of Public Health Management. 2015, 31(2); 223-225 |
| 48 | Jiang Qiuyu | Chinese Journal of Nephrology. 1999, 15(5); 318 |
| 49 | Xu Zaofa | Chinese Journal of Public Health. 1996; 232-233 |
| 50 | Wang Renqun | Modern preventive medicine. 2005, 32(12); 1587-1588 |
| 51 | Luo Lei | Industrial Hygiene and Occupational Diseases. 2021, 47(3); 89-99 |
| 52 | Li Min | Journal of Environment and Health. 2019, 36(7); 607-611 |
| 53 | Lei Huang | Environment international. 2019. 122:301-309 |
| 54 | Xiangfen Cui | Environmental Science and Pollution Research. 2018. 25(15):15089-15101 |
| 55 | Di Zhao | Environmental Science & Technology. 2017. 20;51(12):6756-6764 |
| 56 | HuiFang Luo | Environmental Science and Pollution Research. 2017. 24(2):2047-2054 |
| 57 | Yanhua Gao | International Archives of Occupational and Environmental Health. 2016. 89(7):1137-45 |
| 58 | Dongyue Wang | Chemosphere. 2016. 147:3-8 |
| 59 | Shen Ke | BMC Public Health. 2015. 14;15:656 |
| 60 | Jia Hu | Plos one. 2014. 23;9(12): e115794 |
| 61 | Qi Wang | Plos one. 2014. 9(2): e87817 |
| 62 | WenLi Zhang | Science of the total environment. 2014. 470-471:224-8 |
| 63 | Chen Zhang | Toxicology and Applied Pharmacology. 2013. 271(1):78-85 |
| 64 | Lijian Lei | Toxicology and Applied Pharmacology. 2012. 265(3):373-9 |
| 65 | Yihuai Liang | Environmental Health Perspectives. 2012. 120(2):223-8 |
| 66 | X Ding | Occupational Medicine. 2011. 61(4):277-9 |
| 67 | Xiuli Chang | Experimental Biology and Medicine. 2009. 234(6):666-72 |
| 68 | Xunwei Wu | Environmental research. 2008. 108(2):233-8 |
| 69 | Liang Chen | Diabetes care. 2006. 29(12):2682-7 |
| 70 | Liang Chen | Toxicological sciences. 2006. 91(1):104-12 |
| 71 | G. F. Nordberg | Toxicology and Applied Pharmacology. 2005. 206(2):191-7 |
| 72 | Yujing Cui | Environment international. 2005. 31(6):784-90 |
| 73 | Taiyi Jin | Biometals. 2004. 17(5):513-8 |
| 74 | Xiaodong Wang | Archives of Environmental Health. 2004. 59(6):324-30 |
| 75 | Taiyi Jin | Biometals. 2002. 15(4):397-410 |
| 76 | Xiao Chen | Biological trace element research. 2018. 186(1):114-121 |
| 77 | Xiao Chen | Plos one. 2018. 13(4): e0195682 |
| 78 | Jieying Zhang | Human and Ecological Risk Assessment. 2016. 22(5) |
| 79 | S Cai | Biomedical and environmental sciences. 1992. 5(2):130-5. |
| 80 | Xinru Wang | Scientific Reports. 2020. 10(1):10121. |
| 81 | Yuting Li | BMC Nephrology. 2020. 21(1):385 |
| 82 | Juan Chen | Science of the total environment. 2020. 698:134106 |
| 83 | Peiwei Xu | Chemosphere. 2020. 241:125081 |
| 84 | Peiwei Xu | Environmental Science and Pollution Research. 2021 |
| 85 | Yuanyuan Jin | Environmental Toxicology and Pharmacology. 2020. 79:103381. |

***Table S4*.** Study-ID and corresponding references for urinary cadmium (U-Cd) and N-acetyl-β-glucosidase (NAG).

| Study-ID | First author | Journal and Volume number |
| --- | --- | --- |
| 1 | Liu Zhanqi | China public health. 2000, 16(2); 130-131 |
| 2 | Li Jiqiang | China public health. 2001, 17(6); 486-488 |
| 3 | Zhang Wenli | Journal of Hygiene Research. 2015, 44(5); 780-787 |
| 4 | Zhang Jieying | Journal of Environmental Hygiene. 2015, 5(1); 39-43 |
| 5 | Li Yanfei | Guangxi Medical Journal. 2016, 38(4); 514-516 |
| 6 | Li Hao | Journal of Public Health and Preventive Medicine. 2014, 25(6); 13-16 |
| 7 | Wu Ye | Occupation and Health. 2016, 32(7); 960-962 |
| 8 | Liu Zhanqi | China public health. 2001, 17(10); 923-924 |
| 9 | Jin Taiyi | Chinese journal of environmental & occupational medicine. 2002, 19(1); 10-16 |
| 10 | Wang Keyue | Journal of Environmental & Occupational Medicine. 2011, 28(12); 738-740 |
| 11 | Jiang Qiuyu | Chinese Journal of Nephrology. 1999, 15(5); 318 |
| 12 | Wang Keyue | Labor medicine. 1997, 14(4); 206-207 |
| 13 | Wang Renqun | Modern preventive medicine. 2005, 32(12); 1587-1588 |
| 14 | Hong Feng | Chinese journal of industrial hygiene and occupational diseases. 2003,21(6); 432-436 |
| 15 | Shang Qi | Journal of environment and health. 1995; 1-3 |
| 16 | Wang Zaijuan | Modern preventive medicine. 2005, 32(12); 1587-1588 |
| 17 | Xiangfen Cui | Environmental Science and Pollution Research. 2018;25(15):15089-15101 |
| 18 | HuiFang Luo | Environmental Science and Pollution Research. 2017;24(2):2047-2054 |
| 19 | Yihuai Liang | Environmental Health Perspectives. 2012;120(2):223-8 |
| 20 | G F Nordberg | Toxicology and Applied Pharmacology. 2005;206(2):191-7 |
| 21 | Yujing Cui | Environment International. 2005;31(6):784-90 |
| 22 | Taiyi Jin | Biometals. 2004;17(5):513-8 |
| 23 | Jieying Zhang | Human and Ecological Risk Assessment: An International Journal. 2016; 1-27 |
| 24 | Yuting Li | BMC Nephrology. 2020;21(1):385 |
| 25 | Juan Chen | Science of the Total Environment. 2020, 698:134106 |
| 26 | Peiwei Xu | Chemosphere. 2020, 241:125081 |
| 27 | Lei Huang | Environment International. 2019, 122:301-309 |
| 28 | Peiwei Xu | Environmental Science and Pollution Research. 2021 |
| 29 | Yuanyuan Jin | Environmental Toxicology and Pharmacology. 2020, 79:103381. |

***Table S5*.** Study-ID and corresponding references for urinary cadmium (U-Cd) and microalbumin (mALB).

| Study-ID | First author | Journal and Volume number |
| --- | --- | --- |
| 1 | Kang Hui | Journal of Environmental & Occupational Medicine. 2018, 35(3); 196-202 |
| 2 | Zhou Ni | Modern Preventive Medicine. 2013, 40(8); 1485-1486 |
| 3 | Wang Keyue | Journal of Environmental & Occupational Medicine. 2011, 28(12); 738-740 |
| 4 | Wang Renqun | Journal of environment and health. 2006, 23(3); 202-204 |
| 5 | Chen Liang | Chinese journal of industrial hygiene and occupational diseases. 2006,24(1); 7-11 |
| 6 | Hong Feng | Chinese journal of preventive medicine. 2004, 38(6); 374-378 |
| 7 | Hong Feng | Chinese journal of industrial hygiene and occupational diseases. 2003,21(6); 432-436 |
| 8 | Jin Taiyi | Chinese journal of environmental & occupational medicine. 2002, 19(1); 10-16 |
| 9 | Huang Bo | Journal of labour medicine. 2000, 17(2);78-80 |
| 10 | Jiang Qiuyu | Chinese Journal of Nephrology. 1999, 15(5); 318 |
| 11 | Wu Xunwei | Chinese Journal of Public Health. 1997, 16(2); 99-101 |
| 12 | A Lata | Chinese Journal of Industrial Medicine. 1992, 5(1); 8-9 |
| 13 | Yihuai Liang | Environmental Health Perspectives. 2012;120(2):223-8 |
| 14 | Xiao Chen | Environmental Toxicology and Pharmacology. 2011;32(1):46-53 |
| 15 | Xunwei Wu | Environmental Research. 2008;108(2):233-8 |
| 16 | Liang Chen | Diabetes Care. 2006;29(12):2682-7 |
| 17 | G F Nordberg | Toxicology and Applied Pharmacology. 2005;206(2):191-7 |
| 18 | Yujing Cui | Environment International. 2005;31(6):784-90 |
| 19 | Taiyi Jin | Biometals. 2002;15(4):397-410 |
| 20 | Taiyi Jin | Biometals. 2004;17(5):513-8 |
| 21 | Xiuli Chang | Experimental Biology and Medicine. 2009;234(6):666-72 |
| 22 | Juan Chen | Science of the Total Environment. 2020, 698:134106 |
| 23 | Yuanyuan Jin | Environmental Toxicology and Pharmacology. 2020, 79:103381. |

***Table S6*.** Study-ID and corresponding references for urinary cadmium (U-Cd) and retinol binding Protein (RBP).

| Study-ID | First author | Journal and Volume number |
| --- | --- | --- |
| 1 | Wu Lin | Infection International. 2020, 9(2); 29 |
| 2 | Chen Lianguang | Occupation and Health. 2013, 29(16); 1972-1973 |
| 3 | Zhou Ni | Modern Preventive Medicine. 2013, 40(8); 1485-1486 |
| 4 | Liu Zhidong | Occupation and Health. 2012, 28(5); 537-538 |
| 5 | Li Li | Practical Preventive Medicine. 2011, 18(12); 2322-2323 |
| 6 | Wen Xianzhong | The journal of practical medicine. 2011, 27(15); 2751-2753 |
| 7 | Shi Xinshan | China occupational medicine. 2010, 37(1); 49-50 |
| 8 | Chen Min | Chinese journal of health laboratory technology. 2008, 18(8); 1535-1536 |
| 9 | Xie Ruilin | China tropical medicine. 2008, 8(4); 550-551 |
| 10 | Chen Caihui | China tropical medicine. 2006, 6(6); 958-959 |
| 11 | Wang Renqun | Journal of environment and health. 2006, 23(3); 202-204 |
| 12 | Wang Renqun | Modern preventive medicine. 2005, 32(12); 1587-1588 |
| 13 | Jin Taiyi | Chinese journal of environmental & occupational medicine. 2002, 19(1); 10-16 |
| 14 | Wang Wenlin | Chinese journal of industrial medicine. 2001, 14(4); 193-196 |
| 15 | Huang Bo | Journal of labour medicine. 2000, 17(2); 78-80 |
| 16 | Xiao Chen | Journal of Applied Toxicology. 2018;38(10):1365-1373 |
| 17 | Xiangfen Cui | Environmental Science and Pollution Research. 2018;25(15):15089-15101 |
| 18 | Xiao Chen | Plos One. 2018;13(4): e0195682 |
| 19 | Yujing Cui | Environment International. 2005;31(6):784-90 |
| 20 | Peiwei Xu | Chemosphere. 2020, 241:125081 |
| 21 | Yuanyuan Jin | Environmental Toxicology and Pharmacology. 2020, 79:103381. |
